# MSLI-Net: retinal disease detection network based on multi-segment localization and multi-scale interaction

**DOI:** 10.3389/fcell.2025.1608325

**Published:** 2025-06-06

**Authors:** Zhenjia Qi, Jin Hong, Jilan Cheng, Guoli Long, Hanyu Wang, Siyue Li, Shuangliang Cao

**Affiliations:** ^1^ School of Information Engineering, Nanchang University, Nanchang, China; ^2^ School of Advanced Energy, Sun Yat-sen University, Shenzhen, China; ^3^ Department of Radiological Sciences, University of California Los Angeles, Los Angeles, CA, United States; ^4^ Department of Radiation Oncology, The Affiliated Cancer Hospital of Zhengzhou University & Henan Cancer Hospital, Zhengzhou, China

**Keywords:** retinal disease detection, multi-scale feature fusion, lesion localization, wavelet transform, noise suppression

## Abstract

**Background:**

The retina plays a critical role in visual perception, yet lesions affecting it can lead to severe and irreversible visual impairment. Consequently, early diagnosis and precise identification of these retinal lesions are essential for slowing disease progression. Optical coherence tomography (OCT) stands out as a pivotal imaging modality in ophthalmology due to its exceptional performance, while the inherent complexity of retinal structures and significant noise interference present substantial challenges for both manual interpretation and AI-assisted diagnosis.

**Methods:**

We propose MSLI-Net, a novel framework built upon the ResNet50 backbone, which enhances the global receptive field via a multi-scale dilation fusion module (MDF) to better capture long-range dependencies. Additionally, a multi-segmented lesion localization module (LLM) is integrated within each branch of a modified feature pyramid network (FPN) to effectively extract critical features while suppressing background noise through parallel branch refinement, and a wavelet subband spatial attention module (WSSA) is designed to significantly improve the model’s overall performance in noise suppression by collaboratively processing and exchanging information between the low- and high-frequency subbands extracted through wavelet decomposition.

**Results:**

Experimental evaluation on the OCT-C8 dataset demonstrates that MSLI-Net achieves 96.72% accuracy in retinopathy classification, underscoring its strong discriminative performance and promising potential for clinical application.

**Conclusion:**

This model provides new research ideas for the early diagnosis of retinal diseases and helps drive the development of future high-precision medical imaging-assisted diagnostic systems.

## 1 Introduction

**FIGURE 1:**
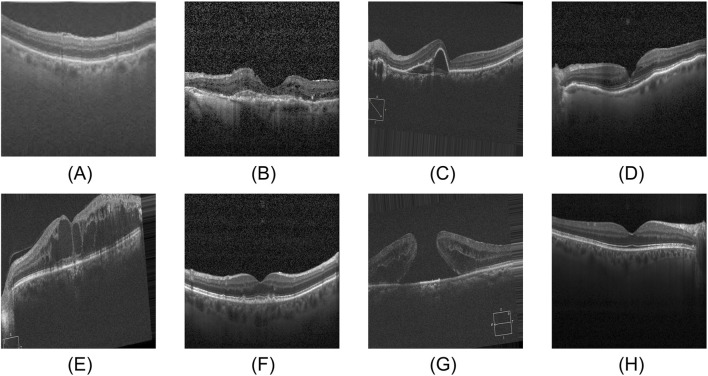
Eight categories of OCT images. **(A)** AMD, **(B)** CNV, **(C)** CSR, **(D)** DME, **(E)** DR, **(F)** DRUSEN, **(G)** MH, **(H)** NORMAL.

The eye plays an indispensable role in how we perceive the world. Its retina, which primarily receives, adjusts, and relays visual stimuli from the environment, supplies the brain with essential visual information, serving as the core structure for our visual perception (Grossniklaus et al., 2015; Kermany et al., 2018). Consequently, retinal diseases are predisposed to causing severe visual impairment and even permanent blindness. At the same time, retinal diseases typically lack pronounced early clinical symptoms, and patients often fail to notice changes in their condition in time, missing the optimal window for intervention. Therefore, early diagnosis combined with high-precision detection is vital in slowing disease progression and minimizing visual impairment (Pennington and DeAngelis, 2016; Robinson, 2003). Figure 1 shows the OCT images of normal retina and seven common retinal diseases.

As a non-contact, non-invasive imaging technique, optical coherence tomography (OCT) utilizes low-coherence interferometry to obtain high-resolution cross-sectional images of biological tissues. With its excellent imaging performance, OCT has become an indispensable diagnostic tool in ophthalmology, playing an increasingly important role in early screening, clinical diagnosis, and efficacy assessment of retinal diseases (Huang et al., 1991). Although this technology has significantly improved the diagnostic efficiency and accuracy of doctors in detecting related conditions, manual interpretation of retinal OCT images still faces considerable challenges in clinical settings. On one hand, as the incidence of retinal diseases continues to rise, the relative scarcity of specialized healthcare resources makes it challenging to meet the ever-growing demand for diagnosis and treatment. On the other hand, the identification of lesion features in OCT images relies heavily on the doctor’s professional knowledge and clinical experience, rendering the diagnostic process highly subjective and potentially compromising diagnostic accuracy (Tsuji et al., 2020; He et al., 2023). In this context, the precise classification of OCT images to distinguish various types of retinal lesions has emerged as an indispensable component in the diagnosis of retinal diseases (Khalil et al., 2024). Therefore, the development of automated retinal image diagnosis systems is essential to assist clinicians in accurately detecting retinal pathologies.

In recent years, deep learning technology has made significant progress in both natural language processing and computer vision, which has promoted the development of various AI-driven diagnostic techniques. Among these, convolutional neural networks (CNN) have increasingly been applied in medical image analysis owing to their excellent feature extraction and pattern recognition capabilities (Zhang et al., 2024; Zhang et al., 2025; Gong et al., 2024; Ji et al., 2022; Simonyan and Zisserman, 2014). Numerous CNN-based models have been developed to address complex tasks such as disease detection (Hong et al., 2019b; Simonyan and Zisserman, 2014), image segmentation (Hong et al., 2022a; Li et al., 2024; Hong et al., 2022b), and classification (Hong et al., 2020a; Hong et al., 2020b; Zhu et al., 2022; Wan et al., 2024; Zhang et al., 2022), substantially enhancing both the automation and diagnostic efficiency in medical image processing. In the realm of ophthalmic image analysis, CNN have been extensively employed for OCT image classification and lesion detection, yielding noteworthy results (Qian et al., 2025; Subramanian et al., 2022; Laouarem et al., 2024; Song et al., 2025). For instance, Qian et al. enhanced the model’s feature representation by fusing the outputs of multiple DenseBlocks based on DenseNet121 and replaced the positive and negative sample pairs in the conventional triplet loss with the class proxy concept. This modification enabled more efficient and accurate classification of retinal OCT images (Qian et al., 2025).

However, the inherent characteristics of OCT retinal images pose a significant challenge to the discriminative performance of existing models. On the one hand, because OCT is a grayscale imaging technique, subtle lesion features are not clear enough to be accurately identified. Moreover, there is a certain diversity in the shape, size and spatial distribution of lesion regions, which also increases the difficulty of lesion localization (Cheng et al., 2025; Simonyan and Zisserman, 2014; Lo et al., 2019). On the other hand, limited by the performance of imaging equipment and hardware conditions, OCT images are often accompanied by unavoidable noise interference during the acquisition process, resulting in many CNN-based models capturing a large amount of speckle noise information while learning lesion features, which makes the model unable to accurately distinguish between important and unimportant features, thus affecting the model’s discriminative ability. In addition, the process of performing downsampling operations to extract high-level semantic features in CNN-based models often inevitably leads to the reduction of the spatial resolution of the image, resulting in the loss of some of the critical lesion information, which together with the residual noise interference weakens the model’s discriminative ability for the lesion region (Zhang et al., 2019; Zhao et al., 2022; Alaba and Ball, 2024).

To address these challenges, this study proposes a retinal disease detection network (MSLI-Net) built upon multi-stage localization and multi-scale interaction. Initially, the network utilizes the residual blocks of ResNet50 for preliminary feature extraction from retinal images. It then employs a Multiscale Dilation Fusion Module (MDF) to enhance the feature representation across scales and expand the model’s receptive field. Subsequently, a Multi-segmented Lesion Localization Fusion Module (LLM) is adopted to emphasize the lesion regions and suppress background noise. Finally, we introduce an MSA module (Xiao et al., 2023) and design a Wavelet Subband Spatial Attention Module (WSSA) to further refine the feature representations in the lesion regions, thereby achieving more precise disease detection. The contributions of this paper are as follows:

1) Our MSLI-Net is based on the ResNet50 network framework, which effectively integrates the MDF, LLM and WSSA, realizing the complementary advantages between shallow high-resolution features and deep semantic information. This enables the model to significantly enhance the representation of lesion regions in retinal OCT images, and effectively improves classification performance.
2) We design a multiscale dilation fusion module (MDF), which effectively extracts multiscale feature information by introducing convolutional branches with different dilation factors and deeply fuses it with original image features. It effectively enhances the global receptive field and improves the model’s ability to model long-range dependencies.
3) We propose a multi-segmented lesion localization fusion module (LLM). By constructing multiple parallel branches, the LLM realizes the hierarchical extraction of local features as well as the enhancement of key channel features of a lesion. This design effectively mitigates the limitations of the traditional channel attention mechanism that is susceptible to interference in the context of complex noise while enhancing the accurate localization of the lesion region.
4) We develop the wavelet subband spatial attention module (WSSA) based on the introduction of the MSA module. This module decomposes the input features into four subbands of different frequencies by discrete wavelet transform, and realizes feature interaction and information fusion across subbands. The module is capable of extracting lesion-related features in greater detail while effectively suppressing noise interference.
5) We evaluate our model on the publicly available OCT-C8 dataset, achieving 96.72% accuracy in retinal OCT classification, demonstrating that this model has a strong discriminative capability in this domain.

## 2 Related work

In recent years, the advancement of deep learning technology and its extensive application in medical image analysis have propelled research and produced significant results in fundus image analysis (Zheng et al., 2024; Xu et al., 2022a; Xu et al., 2022b). Early studies mainly focused on transfer learning and architecture optimization for classical convolutional neural networks (CNN). Wang et al. employed a transfer learning strategy by fine-tuning various classical CNN models (including VGG16, ResNet18, ResNet50, and InceptionV3) that were pre-trained on the ImageNet dataset, thereby achieving higher precision in retinal OCT image classification (Wang et al., 2019). Meanwhile, steady progress has been made in refining the model architecture itself, such as Karthik et al., who proposed Edgen blocks to replace the residual connection method in the traditional ResNet50 and designed a novel activation function to further enhance the network’s ability to capture image boundary features and effectively highlight key lesion information (Karthik and Mahadevappa, 2023). Sunija et al. also designed OCTnet based on the ResNet50 architecture, achieving excellent classification performance while significantly reducing the number of model parameters (Sunija et al., 2021).

In addition to optimizing traditional CNN architectures, recent research has also focused on fusing CNN and Transformer architectures to further enhance the model’s feature representation and global modeling capabilities. Laouarem et al. proposed a hybrid model, HTC-Retina, that combines the advantages of CNN in local feature extraction with the capability of a visual Transformer for global dependency modeling, effectively overcoming the limitations of a single architecture in image analysis (Laouarem et al., 2024). Similarly, the CRAT network mitigates the common attention collapse problem in deep Transformers by introducing the Re-Attention module to dynamically adjust the multi-head self-attention mechanism (Yang et al., 2025). Moreover, the introduction of the Swin Poly transformer network further broadens the research boundaries of fusion modeling, and its mechanism of establishing flexible connectivity between image regions significantly improves the model’s ability to facilitate information exchange among multi-scale features (He et al., 2023).

In addition to integrating different model architectures, task-level co-design has emerged as a prominent research topic. Diao et al. proposed an innovative method that tightly integrates segmentation and classification tasks (Diao et al., 2023). This approach employs an auxiliary segmentation branch within the classification network (CM-CNN) to generate a complementary mask for the input image, which is subsequently used to enhance the original features and effectively guide the classification network to focus on the features of the lesion region, thereby improving classification performance. Moreover, the application of the Grad-CAM algorithm enables CM-CNN to generate a class activation map (CAM) that further assists the segmentation network (CAM-UNet) in refining its segmentation accuracy, ultimately achieving more precise feature extraction and segmentation of the lesion regions. This model exhibits excellent performance on both classification and segmentation tasks, demonstrating the potential of explicit information interaction between tasks in enhancing diagnostic performance.

### 2.1 Image cropping and local feature extraction

It has been shown that the classification performance of deep learning models can be effectively improved by an appropriate cropping strategy for retinal OCT images (Awais et al., 2017). Some researchers have manually cropped out rectangular boxes containing lesion regions at the image preprocessing stage to help neural networks capture key lesion features more effectively (Kaothanthong et al., 2023). However, this cropping method is not only cumbersome and time-consuming, but also poses the risk of degrading the model performance by mistakenly deleting important lesion features. To address these issues, some studies have proposed dividing the OCT images into fixed-size patches and extracting features from each patch individually, thereby improving the model’s ability to extract features from local lesion regions while preserving the overall spatial structure of the image (Dutta et al., 2023).

In other related studies, Sharma et al. proposed a network structure called AELGNet, which successfully achieved efficient capture of both subtle and global features of plant leaf images by partitioning the image feature map into four fixed patches and extracting local features using independent RSA and RCA mechanisms, respectively (Sharma and Vardhan, 2025). However, since most retinal lesions tend to be concentrated in a few localized regions of the image, dividing the patches in a fixed manner and indiscriminately extracting features not only wastes computational resources but also amplifies irrelevant background noise, thereby reducing the model’s classification accuracy.

Unlike existing methods, our proposed LLM employs parallel cropping branches based on the characteristics of retinal OCT images, allowing the model to automatically locate the lesion region and extract key features, effectively mitigating interference from background noise and thereby improving the model’s discriminative capacity and robustness.

### 2.2 Discrete wavelet transform

As a common method for image denoising, the discrete wavelet transform can decompose the signal into low-frequency subbands and high-frequency subbands (Gao and Yan, 2010). Specifically, the low-frequency subbands mainly retain the color and structural information of the image, while the high-frequency subbands preserve detailed features such as edges, textures, and high-frequency noise. This property renders the wavelet transform particularly advantageous in image processing (Yu et al., 2025; Burrus et al., 1998; Xu et al., 2020). Some researchers have attempted to eliminate the HH subband, where the noise is most concentrated, and have introduced an attention mechanism solely for the remaining three subbands, employing the wavelet transform as a downsampling operation to mitigate noise interference (Zhao et al., 2022). Alaba et al. strengthened the information of the LL subband by efficiently fusing the important features in the LH and HL subbands and passing them to the LL subband (Alaba and Ball, 2024). Finder et al. enhanced the model’s receptive field by implementing multi-level wavelet decomposition and independently processing the LL subband (Finder et al., 2024). Although these methods have improved the performance of the wavelet transform in image analysis to some degree, most studies have focused only on low-frequency information or have achieved feature extraction and denoising at the expense of discarding high-frequency information, thereby limiting the comprehensive utilization of the potential information contained in all subbands.

To this end, we propose the WSSA, which synergistically processes all subbands while fully preserving all subband features, adaptively suppressing background noise and highlighting key edge and structural information. Unlike previous approaches that focus solely on information from a single subband, WSSA enables synergistic processing and information interaction between the low-frequency and high-frequency subbands, thereby more comprehensively enhancing the model’s performance in noise suppression and lesion perception.

### 2.3 Dilated convolution

The convolutional kernel is a core component of convolutional neural networks (CNN), but when expanding the receptive field, traditional methods often require stacking multiple convolutional layers into a deep network, which significantly increases the number of parameters. To address this issue, Yu et al. proposed achieving an exponential expansion of the receptive field by introducing different dilation factors, so that the receptive field expands exponentially while parameters grow only linearly (Yu and Koltun, 2015). Based on this design concept, some researchers proposed parallel dilated convolution modules for more efficient image processing tasks (Feng et al., 2020; Li et al., 2019; Bui et al., 2024). For example, Kamran et al. replaced the traditional 3 × 3 convolution with two parallel dilated convolutions with a dilation factor of 2 in the residual block to enhance the network’s ability to model contextual information (Kamran et al., 2019), and Li et al. extracted spatial features from the feature map using a parallel dilated convolution module (Li et al., 2019). Although these designs expanded the receptive field, they did not fully consider the fusion mechanism with the original feature map, which resulted in the loss of local details. In contrast, the MDF designed in our work further strengthens fusion with the original feature map while employing dilated convolution to capture multi-scale features and enlarge the receptive field, thereby preserving local details and enhancing the model’s ability to capture long-range dependencies. Experimental results show that the network using the MDF outperforms the traditional design employing a single dilated convolution module in terms of accuracy.

## 3 Methods

### 3.1 Overall framework

**FIGURE 2:**
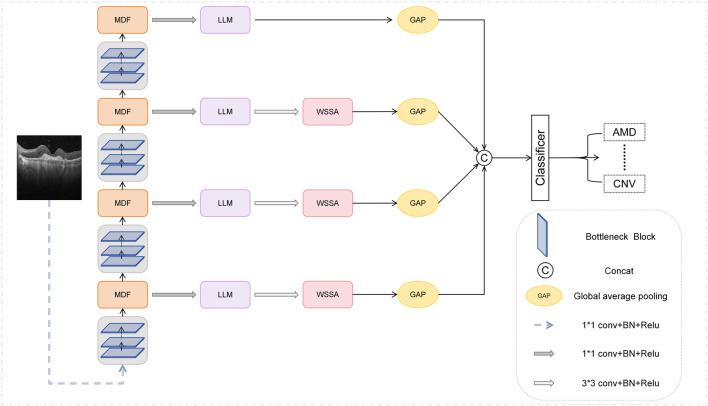
Overall architecture of MSLI-Net.

We propose a new network structure MSLI-Net as shown in Figure 2, the overall architecture of MSLI-Net is composed of three core modules, namely, the multi-scale dilation fusion module (MDF), the multi-segmented lesion localization fusion module (LLM), and the wavelet subband spatial attention module (WSSA). MSLI-Net comprehensively extracts lesion features, effectively enhances focus on key pathological regions, and improves classification accuracy and discriminative performance for retinal OCT images.

Specifically, we feed the image into the multi-scale dilation fusion module after initial feature extraction at various stages of the ResNet50 network. This module extracts multi-scale feature representations with enlarged receptive fields through dilated convolutions with different dilation factors and effectively fuses them with the original feature maps, thereby better incorporating both global semantic context and local detailed features present in the image.

On this basis, we introduced a feature pyramid network (FPN) structure removing the upsampling branches, used the MDF outputs as inputs for each FPN branch, and designed a Multi-segmented Lesion Localization Fusion Module to realize the refinement of the feature maps output from the MDF. In view of the inherent characteristics of retinal OCT images, we innovatively introduced the strategy of parallel cropping in this module to retain and extract feature information segment by segment, which effectively enhanced the localization ability of the lesion region.

Meanwhile, we introduce the MSA module and design the wavelet subband spatial attention module. Considering that when the feature map size is odd, the structural distortion may be caused by the wavelet transform and its inverse transform, the WSSA only process the feature maps produced by the LLM in the first three branches of the FPN. This module effectively enhances key edge information through inter-subband feature interaction and fusion, while suppressing irrelevant noise interference.

Finally, we perform global average pooling on the feature maps output from each of the four branches of the FPN to reduce the spatial dimensionality, and subsequently perform stacked fusion on them in terms of channel dimensions to achieve deep interaction and complementary information between features at different scales. We then feed the fused feature maps into a classifier for retinal image classification. Through this strategy, our network fully fuses the local detail information carried by the high-resolution shallow feature maps with the global semantic information expressed by the deep feature maps, thereby strengthening multi-scale contextual relevance and improving classification accuracy and model robustness.

### 3.2 Multi-scale dilation fusion module (MDF)

To obtain a larger receptive field without reducing the spatial resolution of the feature maps, Yu et al. proposed achieving this by introducing different dilation factors—that is, by effectively increasing the spacing between values in the convolution kernel (Yu and Koltun, 2015; Song et al., 2024). However, due to its sparse sampling pattern resembling a checkerboard, it is prone to triggering the grid effect, which leads to the loss of local information and affects the completeness of feature expression (Mehta et al., 2018). In order to fuse global and local features more effectively, this paper proposes a multi-scale dilation fusion module (MDF). This module effectively improves the overall performance of the model by fully fusing the features extracted by the convolution with different dilation factors with the original features.

**FIGURE 3:**
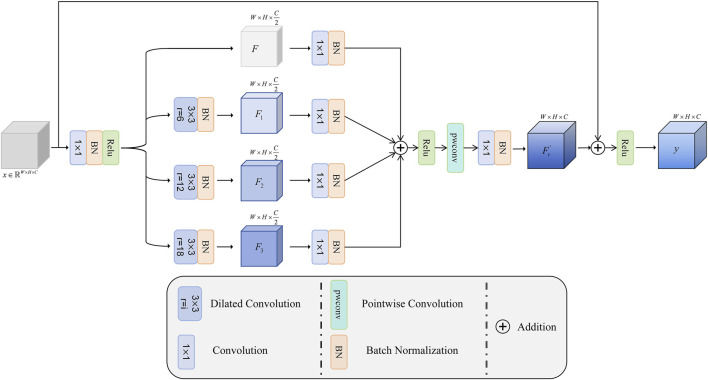
Overall architecture of MDF.

The structure of the MDF is shown in Figure 3. Let the input feature map be $x\in R^{W\times H\times C}$ , where W , H and C denote the width, height and number of channels of the feature map, respectively. First, MDF obtains the new intermediate feature map $F\in R^{W\times H\times \frac{C}{2}}$ by channel compression of the input feature map x . The computational process is shown in Equation 1.

$$F=\mathrm{Relu}\left(\mathrm{BN}\left({\mathrm{Conv}}_{1\times 1}\left(x\right)\right)\right)$$ (1)

Subsequently, different dilation factors (6, 12, and 18) are used to perform convolution operations on F to extract multi-scale context features, which are denoted as $F_{i}\in R^{W\times H\times \frac{C}{2}}$ . To enhance the complementarity between features at different scales, each branch of the extracted feature $F_{i}$ is passed through a 1 × 1 convolutional layer and a batch normalization (BN) layer, to unify the feature scales and adjust the weights to obtain $F_{i}^{\prime }$ , and at the same time, the same operation is performed on the feature map F to obtain $F^{\prime }$ The computational process is shown in Equations 2, 3.

$$F_{i}^{\prime }=BN\left(Conv_{1\times 1}\left(BN\left(Dc_{i-j}\left(F\right)\right)\right)\right)$$ (2)

$$F^{\prime }=\mathrm{BN}\left({\mathrm{Conv}}_{1\times 1}\left(F\right)\right)$$ (3)

where ${\mathrm{Dc}}_{i–j}$ is the inflated convolution with convolution kernel size 3 × 3 and dilation factor j used in branch i . Subsequently, the branch features are fused and the ReLU activation function is introduced to enhance the nonlinear representation, and the fused feature map $F_{r}$ is obtained. The computational procedure is shown in Equation 4.

$$F_{r}=\mathrm{Relu}\left(\sum_{i=1}^{3}F_{i}^{\prime }+F^{\prime }\right)$$ (4)

Next, the fused features are linearly combined between channels by pointwise convolution, so as to further mine the feature relationships between channels and enrich the feature representation. Finally, the number of channels is reduced to the original dimension C and summed elementwise with the input feature map x to finalize the full fusion of features at different scales. The process is shown in Equation 5.

$$y=\text{Re}lu\left(BN\left(Conv_{1\times 1}\left(PWconv\left(F_{r}\right)\right)\right)+x\right)$$ (5)

### 3.3 Multi-segmented lesion localization fusion module (LLM)

**FIGURE 4:**
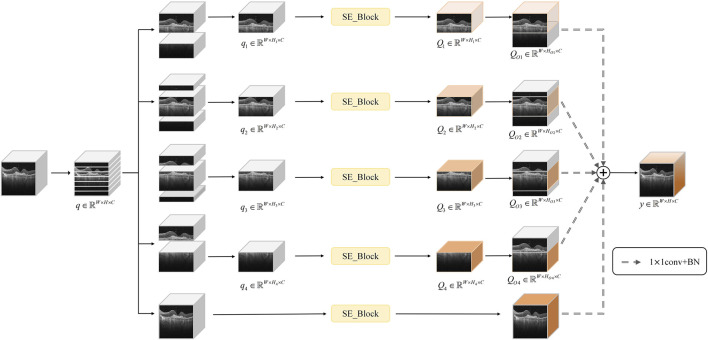
Overall architecture of LLM.

Considering that, in OCT images, the retina typically appears as a horizontally elongated structure while lesions usually occupy only localized regions, we divided the feature map uniformly along the vertical axis into seven subregions and observed that the retinal region is primarily contained within four contiguous segments. To achieve accurate lesion localization, effective extraction of key features, and suppression of irrelevant background noise, this paper proposes a multi-segmented lesion localization fusion module (LLM), as illustrated in Figure 4.

We assume that the original feature map is $q\in R^{W\times H\times C}$ . The LLM divides the feature map into seven subregions along ( H ). The process is shown in Equation 6.

$$H=\left[h_{1},h_{2},h_{3},h_{4},h_{5},h_{6},h_{7}\right]$$ (6)

where H is the height on a single channel of the original feature map and $h_{i}$ is the subregion divided along the height. Then the four consecutive subregions in the feature map are extracted sequentially from top to bottom in each of the four branches to form a subfeature map, i.e., $q\in R^{W\times H_{i}\times C}$ . The specific $H_{i}$ is shown in Equation 7.

$$H_{i}=\left[h_{i},h_{i+1},h_{i+2},h_{i+3}\right]$$ (7)

Subsequently, the SE channel attention mechanism is introduced to process the sub-feature maps of the above four parallel branches with channel-level features, which further enhances the key channel features in each branch, and yields $Q_{I}\in R^{W\times H_{I}\times C}$ . In order to enable the model to more accurately identify which consecutive subregions the lesions are specifically located in, we restore the SE-processed sub-feature maps to their original sizes by re-stitching the sub-feature maps with the discarded portions, i.e., obtaining $Q_{\mathrm{OI}}\in R^{W\times H_{\mathrm{OI}}\times C}$ , and unify the feature scales and adjust the weights through a 1 × 1 convolutional layer and a batch normalization (BN) layer. In addition, in order to more fully realize the complementary advantages of global and local features, and avoid the situation that a small number of images may have incomplete feature extraction due to the local attention mechanism of the model, we additionally add a fifth branch, which directly performs channel-level feature extraction on the original feature map q , and undergoes unified feature scale adjustment and weight fusion operation with the four parallel cropping branches. The process is shown by Equations 8–10.

$$Q_{OI}^{\prime }=BN\left(Conv_{1\times 1}\left(SE\left(Q_{OI}\right)\right)\right)$$ (8)

$$q^{\prime }=BN\left(Conv_{1\times 1}\left(SE\left(q\right)\right)\right)$$ (9)

$$y=\sum_{i=1}^{4}Q_{OI}^{\prime }+q^{\prime }$$ (10)

With this fusion approach, lesion localization is further enhanced, effectively reducing the susceptibility of the traditional channel-attention mechanism to complex background noise interference.

### 3.4 Wavelet subband spatial attention module (WSSA)

As an effective mathematical approach for addressing nonstationary signal decomposition, the wavelet transform can capture information at various frequencies and time positions by adjusting its scale and translation parameters, thereby reflecting the local variation characteristics of a signal. In the context of the commonly used two-dimensional discrete wavelet transform, the Haar wavelet decomposes the input feature map into four subbands via low-pass and high-pass filters, which correspond to the low-frequency subband (LL), horizontal high-frequency subband (LH), vertical high-frequency subband (HL), and diagonal high-frequency subband (HH). Among these, the low-frequency subband encapsulates the image’s color and structural information, while the high-frequency subbands contain abundant detail and texture information. Subsequently, the signal is then reconstructed through the inverse wavelet transform. During reconstruction, wavelet-based edge detection is first applied to enhance edge features in each subband, then thresholding is performed to eliminate noise.

**FIGURE 5:**
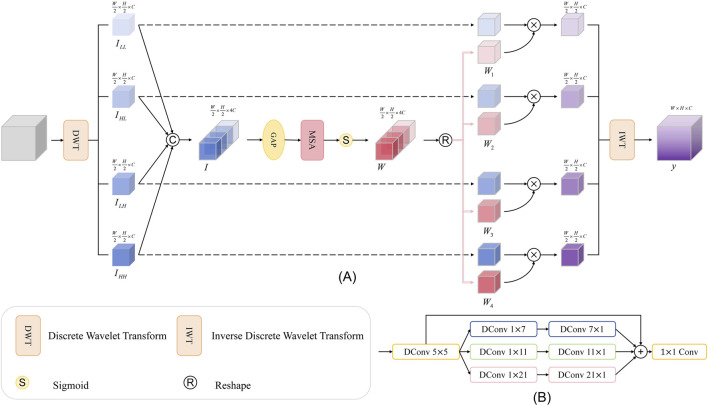
**(A)** Overall architecture of WSSA; **(B)** Architecture of MSA.

However, for retinal OCT images, due to their inherent speckle noise characteristics, it is difficult to effectively denoise them by simply using traditional wavelet transform methods. Therefore, to further suppress noise interference and highlight edge features, we propose the wavelet subband spatial attention module (WSSA) on the basis of the multiscale attention (MSA) module, which is structured as shown in Figure 5A.

In this module, we first use the wavelet transform to decompose the original feature map at multiple scales, and obtain four subbands containing low-frequency and high-frequency information, $I_{\mathrm{LL}},I_{\mathrm{LH}},I_{\mathrm{HL}},I_{\mathrm{HH}}\in R^{\frac{W}{2}\times \frac{H}{2}\times C}$ . In order to realize more efficient collaborative modeling and information interaction between different frequency bands, we stack the four subbands along the channel dimensions, and construct the feature map, $I\in R^{W\times H\times 4C}$ . The specific process is in Equation 11:

$$I=\mathrm{Concat}\left(I_{LL},I_{LH},I_{HL},I_{HH}\right)$$ (11)

Next, we apply an average pooling operation (AvgPool) to the fused feature map I to obtain the smoothed feature map $I_{a}$ . To further enhance the global dependency modeling capability of the features, we introduce the Multihead Self-Attention Mechanism (MSA) to mine the long-distance dependencies and enhance the feature representation capability. Figure 5B shows the MSA, and Equation 12 gives its mathematical expression.

$$\mathrm{Att}={\mathrm{Conv}}_{1\times 1}\left(\sum_{i=0}^{3}MultiChi_{i}\left(DConv_{5\times 5}\left(I_{a}\right)\right)\right)$$ (12)

Where $MultiChi_{i}$ denotes the four feed-forward paths illustrated in Figure 5B, and ${\mathrm{DConv}}_{5\times 5}$ denotes a depthwise convolution with a 5 × 5 kernel (Xiao et al., 2023). The feature map Att obtained after processing by this module is then used to generate the attention weight map $W_{q}\in R^{W\times H\times 4C}$ by the sigmoid activation function. Subsequently, we re-divide $W_{q}$ along the channel dimension into four sub-modules $W_{\mathrm{qi}}\in R^{W\times H\times C}$ . We then multiply each $W_{\text{qi}}$ with its corresponding initial wavelet subband and perform inverse wavelet transform to obtain the output feature map. The specific process is in Equation 13:

$$y=IWT\left(I_{LL}\times W_{q1},I_{LH}\times W_{q2},I_{HL}\times W_{q3},I_{HH}\times W_{q4}\right)$$ (13)

This module enables the global structural information embedded in the low-frequency subbands to effectively guide the recognition of edge details in the high-frequency subbands, effectively suppressing noise interference. At the same time, the fine-grained edge features captured by the high-frequency subbands feed back to the low-frequency subbands, enhancing their ability to perceive the edge region.

## 4 Results and discussion

### 4.1 Datasets

We use the publicly available OCT-C8 dataset (Obuli, 2021) to evaluate the performance of the model proposed in this paper. The dataset contains a total of 24,000 optical coherence tomography (OCT) images of seven types of retinal diseases as well as normal retina: Age-related Macular Degeneration (AMD), Choroidal Neovascularization (CNV), Central Serous Retinopathy (CSR), Diabetic Macular Edema (DME), Diabetic Retinopathy (DR), Yellow deposits under the retina (Drusen), Macular Hole (MH), and Healthy eyes with no abnormalities (NORMAL). Each category contains 3,000 images. The official data split is 2,300 images for training, 350 for validation, and 350 for testing. Considering that increasing the training sample size can improve the model generalization ability, in this paper, the original training set and the validation set are combined as the training set (2,650 images) for model training, and the test set (350 images) remains unchanged.

### 4.2 Evaluation metrics

To evaluate the effectiveness of the model, we use Accuracy (ACC), Precision, Sensitivity, and F1-score as the classification metrics. The formulas for these metrics are as follows.

$$\mathrm{Accuracy}=\frac{n}{N}$$ (14)

$$\mathrm{Precision}=\frac{\mathrm{TP}}{TP+FP}$$ (15)

$$\mathrm{Sensitivity}=\frac{TP}{TP+FN}$$ (16)

$$F1=\frac{2\times \mathrm{Precision}\times \mathrm{Sensitivity}}{\mathrm{Precision}+\mathrm{Sensitivity}}$$ (17)

where n denotes the number of samples in the test set whose classifier prediction results match the true labels, and N is the total number of samples in the test set. In addition, the symbols TP , FP , and FN used in Equations 14–17 denote the number of samples that are true positive (both the actual label and the classification result are in the positive class), false positives (the true label is in the negative class while the classifier predicts the positive class), and false negatives (the true label is in the positive class but the classifier predicts the negative class), respectively.

### 4.3 Implementation details

The training and testing of this experiment were done on a single NVIDIA RTX 4090 GPU. In the data preprocessing stage, we uniformly resize the input feature map to 224 × 224 pixels and normalize the image using pre-calculated mean and standard deviation. During the training process, the loss function was chosen to be cross-entropy loss and the model was optimized using the Adam optimizer, where the optimizer parameters were set to $\beta_{1}=0.9$ , $\beta_{2}=0.999$ . The weight decay parameter was set to $1\times 10^{-4}$ to reduce the risk of overfitting. In addition, in this study, the learning rate was fixed to 0.001, the batch size was set to 64, and trained for 60 epochs. In order to improve the training efficiency and reduce the memory consumption, the mixed-precision training technique provided by PyTorch, i.e., autocast and GradScaler, is used in the experimental process. In the performance evaluation of the model, the model weights at the 60th epoch were used for testing, and the experiments are repeated independently under the same experimental conditions for six times, and the average of the results of the six experiments is taken as the performance metrics of the model. The average of the six experimental results was finally taken as the model performance index.

### 4.4 Performance of our proposed method

In this section, we evaluate the classification performance of the proposed MSLI-Net model on the OCT-C8 retinal image dataset and analyze it in comparison with several representative convolutional neural network architectures, including ResNet50 (He et al., 2016), VGG16 (Simonyan and Zisserman, 2014), GoogLeNet (Szegedy et al., 2015), InceptionV3 (Szegedy et al., 2016), DenseNet121 (KQ, 2018) and EfficientNetB3 (Tan and Le, 2019), among others. In addition to the classical architectures, we also compare them with some of the models that have performed well in the retinal OCT image analysis task in recent years, including CTransCNN (Wu et al., 2023), MedViT (Manzari et al., 2023) and MRVM (Zuo et al., 2024). According to the experimental results shown in Table 1, DenseNet121 has the highest accuracy of 96.41% on the OCT-C8 dataset among the compared baseline models, followed by the MRVM model with an accuracy of 96.20%. Our MSLI-Net achieves a classification accuracy of 96.72% and outperformed the other compared models in all metrics. This demonstrates clear superiority in performance.

**TABLE 1:** Performance comparison of the OCT-C8 dataset (%).

Method	Accuracy	Precision	Sensitivity	F1
ResNet50 (He et al., 2016)	95.08	95.34	95.08	95.09
VGG16 (Simonyan and Zisserman, 2014)	95.69	95.77	95.69	95.69
GoogLeNet (Szegedy et al., 2015)	95.86	96.00	95.86	95.84
InceptionV3 (Szegedy et al., 2016)	89.72	90.96	89.72	89.76
DenseNet121 (KQ, 2018)	96.41	96.46	96.41	96.40
EfficientNetb3 (Tan and Le, 2019)	92.57	93.13	92.57	92.52
CTransCNN (Wu et al., 2023)	94.69	94.69	94.69	94.94
MedViT (Manzari et al., 2023)	95.96	95.96	95.96	95.95
MRVM (Zuo et al., 2024)	96.20	96.21	96.21	96.19
MSLI-Net (Ours)	**96.72**	**96.75**	**96.72**	**96.72**

Bold values indicate the best result under each evaluation metric.

**FIGURE 6:**
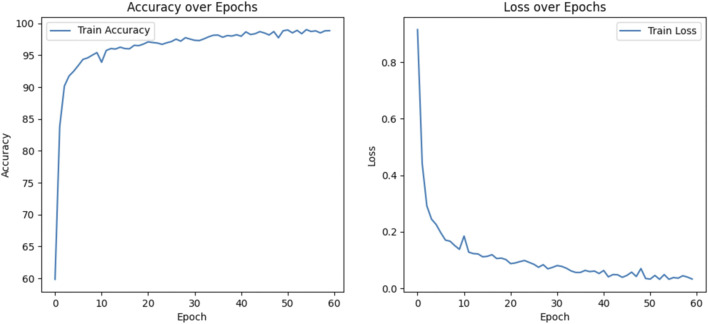
Accuracy and loss during the training process.

**FIGURE 7:**
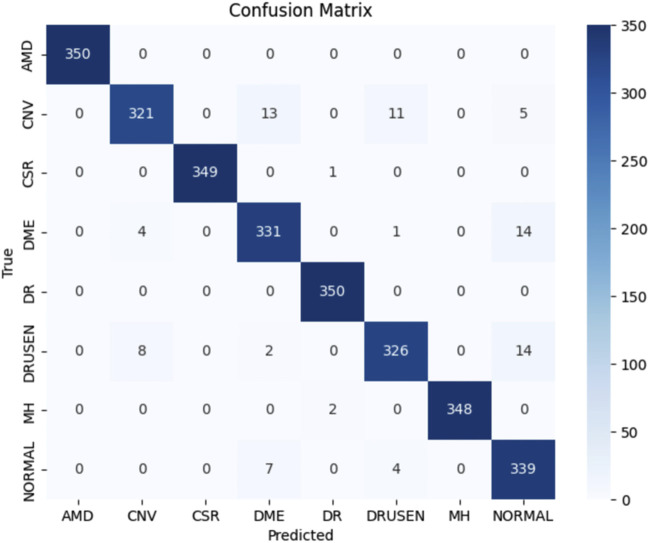
The confusion matrix of the results, with an accuracy of 96.93%.

Figure 6 shows the training metrics for MSLI-Net, where the left graph shows the training-accuracy curve and the right graph shows the training-loss curve. It can be observed that both curves eventually stabilize without significant overfitting. Figure 7 further shows the confusion matrix obtained from one representative experiment. As can be seen, our model achieves 100% classification accuracy on AMD and DR categories, and relatively lower classification accuracy on CNV, DME and DRUSEN, but still maintains a high overall level. These results fully demonstrate the strong generalization capability of MSLI-Net in the task of automatic classification of multi-category retinal OCT images, further validating the effectiveness of the proposed method.

### 4.5 Ablation study

To evaluate the contribution of each module in the proposed model to the overall performance, we conducted systematic ablation experiments on the OCT-C8 dataset, the results of which were shown in Table 2. The accuracy was 95.08% when using ResNet50 alone, which we adopted as our baseline. We first built the ResNet50+MDF architecture by adding the multi-scale dilation fusion module (MDF) to each stage of ResNet50, at which point the model accuracy was improved to 96.04%, an improvement of about 1% from the baseline. Next, we introduced the feature pyramid network (FPN) that removes the up-sampling branches, and added the multi-segmented lesion localization fusion module (LLM) on top of it to form the FPN-ResNet50+MDF + LLM architecture. The results showed that this combination further improves the model accuracy to 96.46%. Finally, we incorporated the Wavelet Subband Spatial Attention module (WSSA) into the first three FPN branches to form the full MSLI-Net; this achieved 96.72% accuracy. These results confirm that each module synergistically enhances overall performance.

**TABLE 2:** Ablation experiment results on OCT-C8 dataset (%).

Method	Accuracy	Precision	Sensitivity	F1
ResNet50	95.08	95.34	95.08	95.09
ResNet50+MDF	96.04	96.09	96.04	96.04
FPN-ResNet50+MDF + LLM	96.46	96.51	96.46	96.46
FPN-ResNet50+LLM + WSSA	95.45	95.61	95.45	95.44
FPN-ResNet50+MDF + WSSA	95.79	95.89	95.79	95.78
MSLI-Net (Ours)	**96.72**	**96.75**	**96.72**	**96.72**

Bold values indicate the best result under each evaluation metric.

To further verify the impact of each module on the overall performance, we removed MDF (FPN-ResNet50+LLM + WSSA) and LLM (FPN-ResNet50+MDF + WSSA) from the full model, respectively, and analyzed their performance in comparison with the complete MSLI-Net model. The experimental results show that after removing MDF and LLM, the classification accuracy of the model is 95.45% and 95.79%, respectively, both of which show a decrease compared with the complete structure. This verifies the key role of each module in the performance improvement. The result further demonstrate that there is a close synergistic dependency between the modules, and the absence of any sub-module will weaken the discriminative ability of the model, thus affecting the overall performance.

In order to verify the effectiveness of the multi-scale dilation fusion module (MDF) proposed in this paper, we reproduced three representative dilated convolution modules and individually replaced the MDF with each of them for comparative experiments. Specifically, we reproduced the proposed dilated feature enhancement module (DFE) designed by Bui et al. (2024); the Multi-scale Context Block (MSCB) proposed by Peng et al. (2023); and the ASPP module (Lo et al., 2019) used by Lo et al. in their work. The experimental results are shown in Table 3, where the model accuracy reached 96.42% when the ASPP was used instead of MDF, 96.20% when MSCB was used, and only 96.08% when MDF was replaced by the DFE module. In contrast, using our proposed MDF within the same network architecture, the model achieved an accuracy of 96.72%. Moreover, our model contained 89.69 million parameters and 33.46 GFLOPs—an increase relative to the MSCB model (46.13 million, 20.58 GFLOPs) but still far smaller than both the DFE (206.67 million, 67.98 GFLOPs) and the ASPP (228.94 million, 72.94 GFLOPs) counterparts. These results demonstrate that our MDF effectively enhances feature extraction and semantic understanding performance while maintaining a lightweight architecture.

**TABLE 3:** Comparison of different inflated convolutional modules on OCT-C8 dataset (%).

Method	Accuracy	Precision	Sensitivity	F1	Params(M)	FLOPs (GFLOPs)
DFE (Bui et al., 2024)	96.08	96.23	96.08	96.08	206.67	67.98
MSCB (Peng et al., 2023)	96.20	96.26	96.20	96.20	46.13	20.58
ASPP (Lo et al., 2019)	96.42	96.48	96.42	96.41	228.94	72.94
MDF(Ours)	**96.72**	**96.75**	**96.72**	**96.72**	**89.69**	**33.46**

Bold values indicate the best result under each evaluation metric.

In addition, to evaluate the multi-segmented lesion localization fusion module (LLM), we devised two comparison schemes: one did not introduce a cropping strategy at all and only used the SE channel attention mechanism (Hu et al., 2018) for feature processing; the other used the cropping strategy proposed by Sharma and Vardhan (2025) in the AELGNet model, i.e., to divided the feature map into four patches using a four-quadrant partitioning strategy, and then applied the SE channel attention mechanism to each patch. The comparison results were shown in Table 4, when only the SE channel attention mechanism was used, the model achieved an accuracy of 96.17%. The accuracy dropped to 95.81% when the AELGNet cropping strategy was used, which may have been due to the fact that retinal structures in OCT images are usually distributed in long horizontal strips, and some patches may contain only background information when dividing the feature map with this cropping strategy. Meanwhile, the four patches were processed indiscriminately during the feature extraction process, which led to the amplification of the interference of irrelevant noise in the background region, and ultimately reduced the effectiveness of the model feature extraction. In contrast, our proposed LLM enabled the model to pay more attention to the features in the retinal region, and as shown in the experimental results in Table 4, the classification accuracy and various indexes of the model when using the LLM were significantly better than those of the comparative methods using the SE module and adopting the cropping strategy in AELGNet, thus verifying the effectiveness of the LLM in the retinal OCT image classification task.

**TABLE 4:** Comparison of different cropping strategies on OCT-C8 dataset (%).

Method	Accuracy	Precision	Sensitivity	F1
Cropping Strategy in AELGNet (Sharma and Vardhan, 2025)	95.81	95.93	95.81	95.80
SE (Hu et al., 2018)	96.17	96.28	96.17	96.17
LLM(Ours)	**96.72**	**96.75**	**96.72**	**96.72**

Bold values indicate the best result under each evaluation metric.

To verify the effectiveness of the proposed wavelet subband spatial attention module (WSSA), we designed two sets of comparison experiments. In the first set of experiments, we replaced the WSSA module, in turn, with the following four methods: (1) using the wavelet transform and its inverse transform (OWT) (Talukder and Harada, 2010) without any processing; (2) adopting the WTConv (Finder et al., 2024) proposed by Finder et al. which involves a convolutional operation for each wavelet subband individually; (3) replicating the WCAM proposed by Alaba and Ball (2024), which is processed by fusing the features of the LH and HL subbands to the LL subband; (4) the MSA (Xiao et al., 2023) applying independently to each wavelet subband, constituting the OWT + MSA.

In addition, our WSSA module stacks all wavelet subbands, extracts subband weights via global average pooling, and then refines these weights using the MSA module. These weights are then multiplied with the original subband features to facilitate inter-subband information interaction. Finally, we perform the inverse wavelet transform to restore the image size. Therefore, we further designed a second set of comparative experiments to comprehensively evaluate the advantages of the WSSA. Specifically, without altering the remaining process, we independently excluded each of the LL, LH, HL, and HH subbands—resulting in four modules referred to as w/o LL, w/o LH, w/o HL, and w/o HH—in which only the remaining three subbands are stacked and processed. This setup allows us to analyze the role of each subband in the process of information fusion.

Table 5 shows the performance comparison results of models using different modules in the first set of experiments for the retinal OCT image classification task. The results show that the classification accuracy of the model using OWT is only 95.92%, indicating that although the wavelet transform possesses some image processing capability, the lack of subsequent feature extraction may lead to the disruption of intrinsic structure, which affects the classification performance. Further, the models using WTconv and OWT + MSA obtain classification accuracies of 96.04% and 96.23%, respectively, indicating that there is a close intrinsic correlation between the wavelet subbands, and it is difficult to effectively tap the potential complementary information of each subband by only performing independent feature extraction, thus restricting the enhancement of the model’s discriminative ability. In contrast, the classification accuracy of the model using WCAM that fuses the LH and HL with the LL subband features is 96.44%, which verifies that the information interaction between the subbands helps to fully mine the feature information.

**TABLE 5:** Comparison of the first set of wavelet strategies on the OCT-C8 dataset (%).

Method	Accuracy	Precision	Sensitivity	F1
OWT (Talukder and Harada, 2010)	95.92	96.01	95.92	95.92
WTConv (Finder et al., 2024)	96.04	96.17	96.04	96.03
WCAM (Alaba and Ball, 2024)	96.44	96.48	96.44	96.43
OWT + MSA	96.23	96.32	96.23	96.22
WSSA (Ours)	**96.72**	**96.75**	**96.72**	**96.72**

Bold values indicate the best result under each evaluation metric.

Table 6 displays the results of the second set of comparative experiments. It can be seen that the model accuracy using the second set of comparison methods (w/o LL, w/o LH, w/o HL, and w/o HH) is distributed between 96.12% and 96.31%, highlighting that the synergistic effect of each wavelet subband in feature extraction is indispensable. The model accuracy reaches 96.72% when using our proposed WSSA. This suggests that omitting any sub-band may degrade feature representation, thereby impairing the model’s overall discriminative performance. The effectiveness of WSSA in the retinal OCT image classification task is also demonstrated.

**TABLE 6:** Comparison of the second set of wavelet strategies on the OCT-C8 dataset (%).

Method	Accuracy	Precision	Sensitivity	F1
w/o LL	96.12	96.21	96.12	96.12
w/o LH	96.31	96.38	96.32	96.31
w/o HL	96.30	96.36	96.30	96.29
w/o HH	96.23	96.34	96.23	96.22
WSSA (Ours)	**96.72**	**96.75**	**96.72**	**96.72**

Bold values indicate the best result under each evaluation metric.

### 4.6 Robustness of the noise processing module

OCT image quality varies significantly because acquisition is affected by external factors such as imaging-equipment performance and ambient-light interference. Some images even contain severe speckle noise. These issues pose major challenges for subsequent image processing and analysis. In order to verify the effectiveness of our WSSA model for the denoising of retinal OCT images, we added a multiplicative scattering noise model (Huang et al., 2019) to the test dataset and used the peak signal-to-noise ratio (PSNR) to measure the noise level. It can be expressed by the following Equation:

$$F\left(x,y\right)=g\left(x,y\right)+g\left(x,y\right)\times u\left(x,y\right)$$ (18)

$$\mathrm{PSNR}=20\times \mathrm{lg}\left(\frac{\mathrm{Max}}{\sqrt{\mathrm{MSE}}}\right)$$ (19)

where, in Equation 18, $g\left(x,y\right)$ denotes the original image undisturbed by noise; $u\left(x,y\right)$ is a set of Gaussian noise obeying a mean of 0 and a variance of s ( $\sigma^{2}$ ), whose variance increases with the increase of the gray value of the image; and $F\left(x,y\right)$ denotes the image obtained after adding the noise. Also in Equation 19, Max is the maximum pixel value of the image and MSE denotes the mean square error between the image with noise and the original image.

**FIGURE 8:**
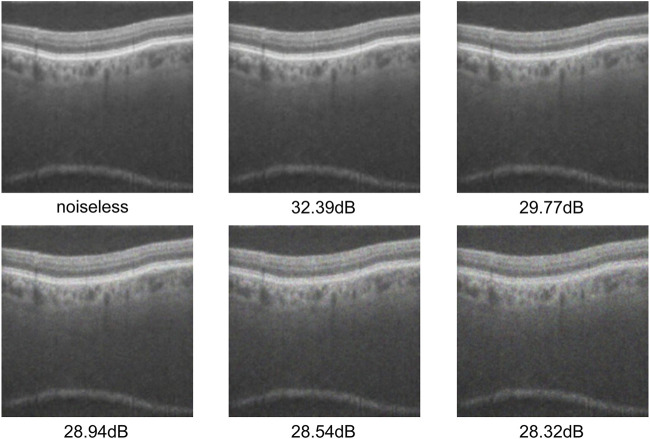
Test dataset after adding different levels of noise.

We trained the model using the original training dataset, and added multiplicative scattering noise of five variance levels ( $\sigma^{2}$ = $0.1^{2}$ , $0.2^{2}$ , $0.3^{2}$ , $0.4^{2}$ and $0.5^{2}$ ) to the test set during the testing phase. These noise intensities correspond to PSNR values of 30.85 dB, 25.22 dB, 21.95 dB, 19.66 dB, and 18.01 dB, respectively. Figure 8 demonstrates the retinal OCT images under different degrees of noise. It can be observed that as noise intensity increases, the PSNR value gradually decreases, the image quality decreases significantly, and the noise interference becomes increasingly pronounced. To verify the robustness of the proposed module in different noise environments, we used the models in Tables 5, 6 for comparative analysis. Tables 7, 8 show the test results of each model under different noise.

**TABLE 7:** Comparison of the first set of wavelet strategies under different noise intensities (dB).

MethodNoise intensity	Noiseless	30.85	25.22	21.95	19.66	18.01
OWT (Talukder and Harada, 2010)	95.92	95.92	95.87	95.66	95.22	94.35
WTconv (Finder et al., 2024)	96.04	95.67	95.94	95.61	95.14	94.5
WCAM (Alaba and Ball, 2024)	96.44	96.44	96.19	96.30	96.00	95.33
OWT + MSA	96.23	95.58	95.64	95.68	95.17	95.07
WSSA (Ours)	**96.72**	**96.77**	**96.68**	**96.62**	**96.32**	**95.82**

Bold values indicate the best result under each evaluation metric.

**TABLE 8:** Comparison of the second set of wavelet strategies under different noise intensities (dB).

MethodNoise intensity	Noiseless	30.85	25.22	21.95	19.66	18.01
w/o LL	96.12	95.82	95.69	95.99	95.19	94.44
w/o LH	96.31	96.31	96.17	96.08	95.56	94.73
w/o HL	96.30	96.28	96.31	96.06	95.88	95.49
w/o HH	96.23	96.08	96.04	95.79	95.57	94.87
WSSA (Ours)	**96.72**	**96.77**	**96.68**	**96.62**	**96.32**	**95.82**

Bold values indicate the best result under each evaluation metric.

As shown in Tables 7, 8, the overall performance of all modules decreases with the increase of noise intensity. Among them, except for the MSLI-Net model proposed in this study and its variant without the HL subband—which both exhibit a performance degradation within 1%—all other models experience a degradation exceeding 1%, specifically ranging from 1.11% to 1.68%. In addition, our model shows significant performance degradation only when the noise intensity decreases to 19.66 dB, and its fluctuation of no more than 0.1% between no noise and a noise intensity of 21.95 dB, demonstrating good stability. Moreover, across all noise levels, the overall performance of our model is always better than that of other comparative methods, indicating that the method in this paper has good robustness under high-intensity noise interference.

In order to verify the robustness of the proposed LLM in noisy environments, we conducted systematic tests on each module listed in Table 4 under different noise intensities, and the test results are shown in Table 9. From the results, it can be seen that the classification accuracy of the model with the AELGNet cropping strategy decreases by 0.55% when noise is first added, which is the largest decrease among the three modules, indicating that the strategy is more sensitive to noise, and further proving that this cropping approach may amplify the interference of extraneous noise in the background region. As noise intensity increases, the performance of the model using only the SE module is more significantly impaired under high-intensity noise. It is worth noting that the model (MSLI-Net) using LLM shows better classification accuracy than the other two strategies across all noise levels, which fully verifies that the LLM method has stronger noise robustness in complex noise environments.

**TABLE 9:** Comparison of the LLM and each module under different noise intensities (dB).

MethodNoise intensity	Noiseless	30.85	25.22	21.95	19.66	18.01
Cropping Strategy in AELGNet (Sharma and Vardhan, 2025)	96.30	95.75	95.69	95.39	95.88	95.49
SE (Hu et al., 2018)	96.23	95.96	95.81	95.58	95.57	94.87
WSSA (Ours)	**96.72**	**96.77**	**96.68**	**96.62**	**96.32**	**95.82**

Bold values indicate the best result under each evaluation metric.

### 4.7 Visualization and analysis

In order to visually assess the effectiveness of the MDF proposed in this paper in multi-scale feature extraction and its ability to deeply integrate with the original image features, this paper introduces the Grad-CAM method to visualize and analyze the image regions that each of the comparative modules pays attention to in the classification decision-making process. The method effectively reveals the ability of each module to pay attention to the key regions of the input image through the generation of heat maps.

**FIGURE 9:**
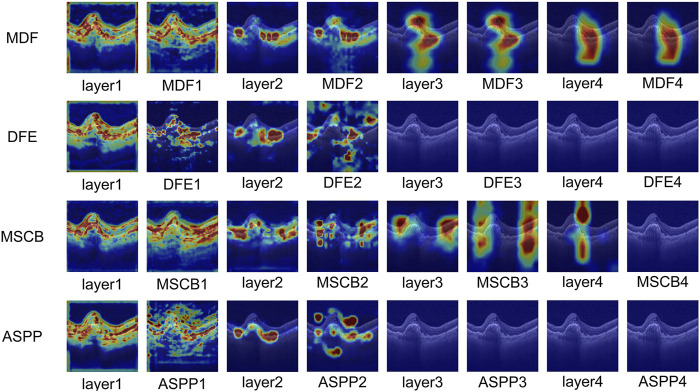
Visualization and analysis of MDF, DFE, MSCB and ASPP.

In this paper, based on each module in Table 3, its corresponding heat map is generated at different network stages of ResNet50 and visualized for comparison. As shown in Figure 9, in the layer1 stage of ResNet50, each module shows high consistency in focusing on the lesion region. As the network deepens, the MDF consistently maintains high consistency with the backbone network at all stages and further enhances its ability to focus on lesion regions. In contrast, during the first two stages, the MSCB consistently aligns its focus on the lesion regions with that of the backbone network and remains relatively stable; however, the region of focus deviates significantly in the third stage. Conversely, DFE and ASPP show a tendency of divergence of the total attention region after the first stage, and although they briefly enhance the ability of layer2 of ResNet50 to focus on the lesion, by the third and fourth stages, their ability to focus on the lesion decreases significantly, and neither of them is able to focus on the lesion portion well.

The comparative results heat map visualization further validates the advantages of the MDF module in feature fusion and semantic modeling. Especially In deeper layers, MDF is still able to maintain a stable and precise attention region, reflecting stronger discriminative and semantic retention abilities.

**FIGURE 10:**
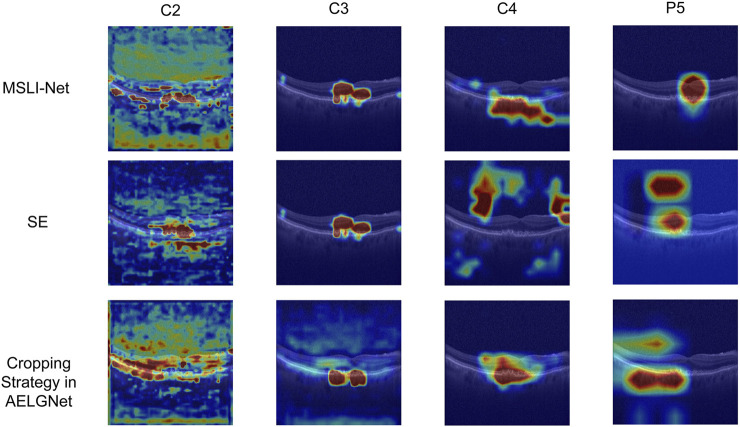
Visualization and analysis of SE, cropping strategy in AELGNet, and LLM.

Meanwhile, we also visualize and compare the mentioned models in Table 4. As shown in Figure 10, in the shallow stage (c2 and c3), the models using SE, the cropping strategy in AELGNet, and the LLM proposed in this paper are able to locate the lesion region more accurately, which reflects a good initial discriminative ability. However, in the deeper network stages (c4 and p5), SE and cropping strategy in AELGNet gradually demonstrate increased background attention, compared to LLM which still maintains a stable focus on the lesion region in the deeper stages. This result fully demonstrates the effectiveness of our LLM’s performance for lesion localization.

## 5 Conclusion

In this study, we propose a novel network architecture called MSLI-Net for the classification task of retinal optical coherence tomography (OCT) images. The model effectively enhances the feature extraction capability of the model for the lesion region and significantly improves the overall classification performance through three core modules, namely, the multi-scale dilation fusion module (MDF), the multi-segmented lesion localization fusion module (LLM), and the wavelet subband spatial attention module (WSSA). On the publicly available OCT-C8 dataset, this method achieves a classification accuracy of 96.72%. We further confirm the critical role of each of the three modules, MDF, LLM and WSSA, in network performance improvement through comprehensive ablation experiments. Meanwhile, MSLI-Net still exhibits robust performance under stronger noise interference environment. MSLI-Net architecture not only has practical value for OCT image classification, but also provides new research ideas and effective technical references for future network design for similar tasks.

## Data Availability

Publicly available datasets were analyzed in this study. This data can be found here: https://doi.org/10.34740/KAGGLE/DSV/2736749.
